# Decoding the function and regulation of the mammalian 12-h clock

**DOI:** 10.1093/jmcb/mjaa021

**Published:** 2020-05-08

**Authors:** Bokai Zhu

**Affiliations:** 1 Aging Institute of UPMC, University of Pittsburgh School of Medicine, Pittsburgh, PA, USA; 2 Pittsburgh Liver Research Center, University of Pittsburgh, Pittsburgh, PA, USA; 3 Division of Endocrinology and Metabolism, Department of Medicine, University of Pittsburgh School of Medicine, Pittsburgh, PA, USA

Both Eastern and Western ancient philosophies posited that a harmony exists between the universe and the human beings (and by extension, all life forms on earth). For example, the Chinese is strongly influenced by the Taoism doctrine of the Unity of Man and Heaven (Raz, 2012), while the great Greek mathematician Pythagoras raised the concept of ‘musica universalis’, which claims the movements of celestial bodies follow mathematical equations and resonate to produce an inaudible harmony of music, of which the harmonious sounds that men make were mere an approximation (Koff and Fiveisky, 1975). The best example of the harmony between the natural world and living organisms probably lies in the well-characterized ∼24-h circadian rhythm, which is synchronized to the diurnal natural cycles of temperature and light coinciding with the 24-h cycle of the Earth self-rotation. Unbeknownst to many, in addition to the circadian rhythms, ∼12-h rhythms also exist in living organisms, with the prominent example of circatidal rhythms found in coastal and estuarine animals that modulate their behavior in tune to the ∼12.4-h ebb and flow of the tides (Wilcockson and Zhang, 2008). Intriguingly, the circatidal rhythms are also evolutionarily conserved in in-land animals that are no longer exposed to tidal cues, including mammals (Zhu et al., 2017, 2018; Pan et al., 2020). In this perspective, I will focus on the mammalian 12-h rhythms and discuss our current understandings of their prevalence, physiological function, and regulation.

## The mammalian 12-h rhythms are prevalent

While the number of 12-h cycling mRNA in mice was originally thought to be relatively small (∼200; Hughes et al., 2009), re-analyzing the same hepatic microarray dataset with a recently developed eigenvalue/pencil method (Antoulas et al., 2018) uncovered a much larger repertoire of 3652 (∼20% of total hepatic mRNA), of which 760 are dominant (whose 12-h amplitudes are the greatest among all identified oscillations) (Zhu et al., 2017). Consistent with the initial analysis, by performing and analyzing a high resolution temporal hepatic RNA sequencing dataset using two orthogonal methods, eigenvalue/pencil and RAIN (Thaben and Westermark, 2014), we uncovered ∼3650 high-confidence 12-h hepatic mRNA (∼27% of all hepatic transcriptome) in mouse liver under constant darkness conditions (Pan et al., 2020). While the prevalence of hepatic 12-h transcriptome matches that of hepatic circadian rhythm (Koike et al., 2012), their amplitudes, on average, are smaller, often in the range of 1.3- to 4-fold changes (Pan et al., 2020). Compared to those of the circadian rhythms, the phases of hepatic 12-h rhythms are more skewed toward CT0 and CT12, corresponding to the dawn and dusk in a daily cycle (Zhu et al., 2017; Pan et al., 2020). This unique phase feature provides valuable clues to the function of mammalian 12-h rhythms, which will be discussed in detail later. Besides transcriptome, 12-h hepatic oscillations are also found abundantly at the proteome and metabolome level (also in the range of 20%–30% of all hepatic proteome and metabolome), which are overrepresented in nucleotide, amino and nucleotide sugar, polyamine, glycerophospholipid, and sphingolipid metabolism pathways (Zhu et al., 2018). Together, the highly coupled 12-h hepatic metabolic gene expression and metabolite oscillations strongly imply the presence of an endogenous 12-h pacemaker that is responsible for the precisely timed orchestration of metabolic flux by temporally regulating the expressions of metabolic enzymes (Pan et al., 2020).

**Figure 1:**
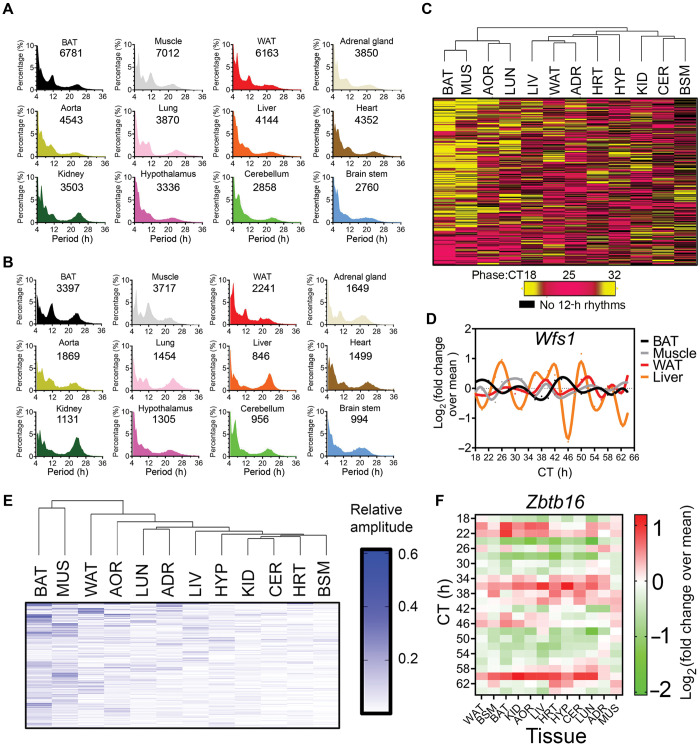
Prevalent 12-h rhythms of gene expression in mouse tissues. Eigenvalue/pencil method was applied to the microarray dataset reported in Zhang et al. (2014). The criterion for defining 12-h oscillation is previously described (Pan et al., 2020). (**A** and **B**) Period distribution of all (**A**) and dominant (**B**) oscillations uncovered in 12 tissues in mice. The number of ∼12-h oscillations in each tissue is shown. (**C**) Hierarchical clustering of the phases of 380 commonly found (in at least eight tissues) 12-h genes. (**D**) *Wfs1* expression in four different tissues. (**E**) Hierarchical clustering of the relative amplitudes of 380 commonly found (in at least eight tissues) 12-h genes. (**F**) Heat map of *Zbtb16* expression.

Are 12-h rhythms also abundant in other tissues in mice? To answer this question, I performed a *post hoc* analysis of a published mouse temporal gene expression atlas (Zhang et al., 2014) and used the eigenvalue/pencil method to identify all ∼12-h mRNA oscillations in 12 different tissues in mice. As shown in Figure 1A and B, 12-h rhythms of gene expression are overall very prevalent in mice, with brown adipose tissue (BAT), skeletal muscle (MUS), and white adipose tissue (WAT) exhibiting the greatest number of both total and dominant 12-h cycling transcripts. On the other end, the three brain tissues, hypothalamus (HYP), cerebellum (CER), and brain stem (BSM), have the least number of 12-h transcripts. I further identified a total of 380 mRNAs exhibiting 12-h rhythms in at least eight tissues, with *Zbtb16* topping the list with 12-h rhythms found in 10 tissues (Figure 1C–F). Hierarchical clustering analysis based upon the phases and amplitudes of these 380 genes revealed distinct 12-h transcriptome signatures associated with different tissues. Particularly, the 12-h transcriptome signatures in BAT and MUS are very similar to each other, but drastically disparate from the rest of the tissues, in terms of both phases and amplitudes (Figure 1C–E). The ‘distinctiveness’ of BAT and MUS’s 12-h transcriptome may be attributed to their unique metabolic profile. Compared to the other organs in mammals, BAT and MUS are highly catabolic with higher levels of fatty acid oxidation and thermogenesis, manifested with high mitochondrial content and increased energy expenditure. In fact, it is believed that BAT and MUS share a common ancestor distinct from WAT (Sanchez-Gurmaches and Guertin, 2014). Therefore, it is plausible to conjecture that the unique 12-h transcriptome signatures in BAT and MUS are hard-wired in their gene regulatory networks during the early developmental stage, which will be further elucidated.

Taken together, I herein demonstrate very prevalent 12-h rhythms of gene expression and metabolism in multiple tissues in mice. Future endeavors should be directed toward charactering the prevalence of 12-h rhythms in primates, including humans, which could be achieved by re-analyzing the diurnal baboon transcriptome data using the eigenvalue/pencil method (Mure et al., 2018), and reconstructing time series human data either using time-of-death information or predicting circadian time computationally as previously described for the identification of circadian rhythms in humans (Anafi et al., 2017; Seney et al., 2019).

## The vehicle-cargo hypothesis on the distinct functions of 12-h vs. the circadian rhythms

**Figure 2:**
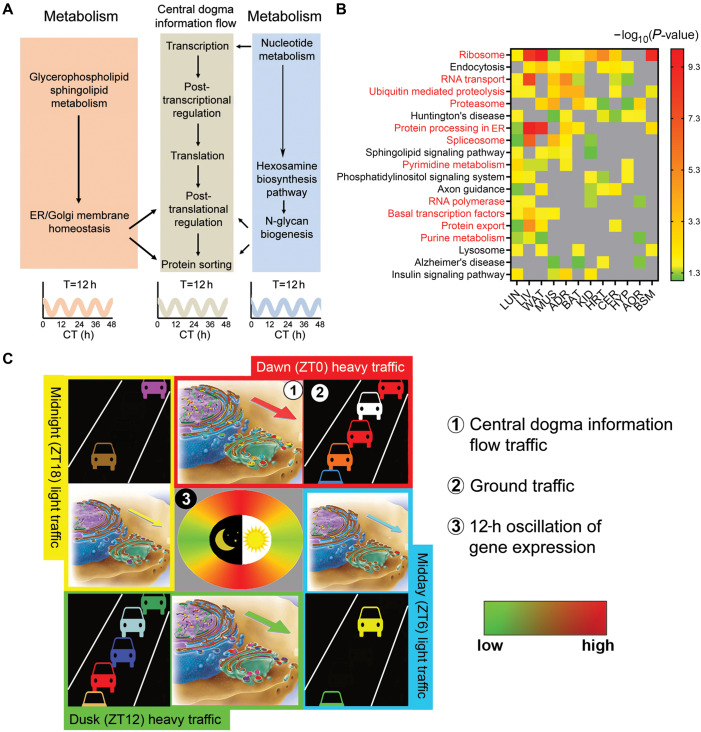
The function of the mammalian 12-h rhythms. (**A**) A diagram showing coordinated 12-h rhythms of gene expression involved in CEDIF and various metabolic pathways. (**B**) Heatmap showing the −log_10_ transformed *P-*values of enriched KEGG GO terms found in 12-h transcriptome in different mouse tissues. Gray means the GO term is not found. (**C**) The vehicle-cargo hypothesis on the distinct functions of 12-h clock vs. the 24-h circadian clock. Adapted from Pan et al. (2020).

So, what is the primary function of the mammalian 12-h rhythms? Even without extensive empirical data, it can be deduced from the functions of the genes exhibiting 12-h rhythms. By performing gene ontology (GO) analysis of global hepatic 12-h transcriptome, we found that the hepatic 12-h transcriptome is strongly enriched in the entire central dogma information flow (CEDIF) process, including mRNA transcription, mRNA processing and export, ribosome biogenesis, translation initiation, and protein folding, processing and sorting in the endoplasmic reticulum (ER) and Golgi, which include both anabolic and catabolic processes (Pan et al., 2020). In addition, we discovered 12-h cycling genes are enriched in numerous metabolic pathways, such as purine and pyrimidine *de novo* synthesis, hexosamine and UDP-GlcNAc biosynthesis, and glycophospholipids and sphingolipids synthesis, consistent with the 12-h hepatic metabolome (Zhu et al., 2017, 2018; Pan et al., 2020). The 12-h rhythm of metabolic gene expression is coupled to that of the CEDIF process (Zhu et al., 2017, 2018; Figure 2A): 12-h rhythm of nucleotide metabolism is essential for 12-h rhythm of transcription and mRNA processing; 12-h rhythm of nucleotide and amino sugar metabolism provides precursors for 12-h rhythm of protein N-linked glycosylation occurring in the ER and Golgi; 12-h rhythms of glycerophospholipid and sphingolipid metabolism contributes to 12-h rhythm of ER/Golgi membrane homeostasis (likely by regulating membrane lipid composition, permeability, and fluidity), the integrity of which is central to protein sorting and vesicle trafficking in the ER and Golgi (McMaster, 2001).

The enriched CEDIF and metabolic gene signatures are not restricted to the liver. As a matter of fact, they are commonly found in the 12-h transcriptomes of all tissues in mice, with the GO term ribosome found enriched in all but two tissues (Figure 2B). In light of these findings, we proposed a vehicle-cargo hypothesis that attempts to decipher the distinct functions of 12-h vs. the circadian rhythms (Pan et al., 2020; Figure 2C). We argue that the 12-h rhythms accommodate demands for increased gene expression/processing at the two ‘rush hours’ by controlling the global traffic capacity (and/or the rate) of the CEDIF (thus the vehicle), in tune to the 12-h cycle of metabolic stress (Zhu et al., 2018). The circadian clock, on the other hand, dictates the particular genes/gene products processed at each rush hour (thus the cargo) as previously suggested (Zhang et al., 2014). An everyday metaphor would be the fluctuating daily traffic on the highway: the 12-h biological rhythm is analogous to the oscillatory operating capacity of the highway, which increases during the two rush hours (e.g. by opening the HOV lane), whereas the function of the circadian clock is likened to determining which cars actually go on the highway at each rush hour. In the majority of the mouse tissues (e.g. liver and adrenal gland), the two ‘rush hours’ correspond to CT0 and CT12, coinciding with the two transition periods between fasting/feeding and sleep/activity at dawn and dusk. At the subjective dawn, the prolonged absence of energy intake combined with reduced but still significant energy expenditure from the subjective night leads to a peak of energy deficiency, while at the subjective dusk, sufficient energy intake during the subjective day combined with reduced energy expenditure gives rise to a peak of energy ‘excess’. Both energy ‘deficiency’ and energy ‘excess’ create greater metabolic stress and can activate the ER-associated unfolded protein response (UPR) to mitigate the original stress (Zhu et al., 2018). In BAT and MUS, these two rush hours appear to shift to midday and midnight, which again likely reflect their unique metabolic profiles.

## Mammalian 12-h rhythms are transcriptionally regulated by a dedicated ‘12-h pacemaker’ involving the ETS, bZIP-containing, and NFY TFs

How are the mammalian 12-h rhythms established at the molecular level? Early studies favor the hypothesis that the mammalian 12-h rhythms are not cell-autonomous and established by the combined effects of circadian clock and fasting-feeding cues (Hughes et al., 2009, 2012; Cretenet et al., 2010). Alternatively, it was suggested that two circadian transcription activators or repressors appearing in anti-phase are theoretically capable of establishing 12-h rhythms of gene expression in a cell-autonomous manner (Westermark and Herzel, 2013). However, through a series of rigorous studies (Zhu et al., 2017, 2018; Antoulas et al., 2018; Pan et al., 2020), our group conclusively demonstrated that the majority of 12-h rhythms are not only independent of the circadian clock, but also cell-autonomous, a finding supported by the observation of a strong overlap between the 12-h transcriptome observed in mouse liver *in vivo* (under constant darkness condition), and in serum-synchronized mouse hepatocyte cell line MMH-D3 *in vitro* (the overlap is more striking when comparing the 12-h transcriptome-regulated biological pathways) (Pan et al., 2020). These findings enticed us to raise the hypothesis that there exists a dedicated and cell-autonomous ‘12-h pacemaker’ responsible for the establishment and maintenance of mammalian 12-h rhythms.

**Figure 3:**
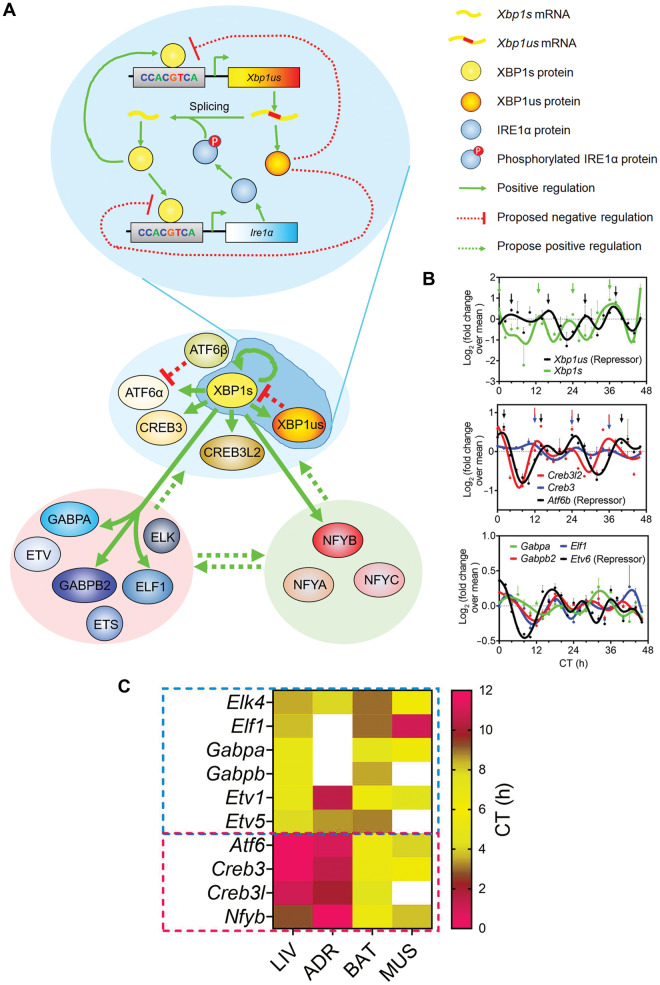
The transcriptional regulatory network of the mammalian 12-h clock. (**A**) A simplified model demonstrating our current understanding of the transcriptional regulation of the mammalian 12-h clock. Solid lines indicate interactions supported by experimental evidence; dashed lines indicate proposed interactions. (**B**) Temporal quantitative PCR (for *Xbp1s* and *Xbp1us*) and RNA sequencing data for key TFs participating in the transcriptional regulation of mammalian 12-h clock. (**C**) Heat map showing the phases of 12-h cycling TFs in four tissues.

In mammals, the 24-h circadian rhythms are established mainly at the transcriptional level by an intricate network of transcription factors (TFs) forming the transcriptional-translational feedback loop (TTFL; Takahashi, 2017). Could a similar mechanism be at play for the 12-h rhythms? If so, I postulate that the core 12-h clock TFs should at least meet the following three criterion: (i) at the mRNA/protein level, these TFs should follow robust 12-h rhythms of expression in tissues with prevalent 12-h transcriptome; (ii) the DNA-binding motifs of these TFs should also be enriched at the regulatory regions of 12-h cycling genes; and (iii) the DNA-binding motifs of these TFs are expected to co-occur *in cis* within close proximity of each other. After rigorous bioinformatics analysis, three top hits: ATF6α, ELK4, and NFYB were identified, although *Zbtb16* has 12-h rhythms observed in all but two tissues, its reported DNA-binding motif TACAGT (Agrawal Singh et al., 2019) was not found enriched at the promoters nor enhancers of 12-h cycling genes. ATF6α belong to the basic leucine zipper (bZIP) family of TFs, which also include ATF6β, XBP1s, CREB3, and CREB3L2, and binds to consensus DNA-binding motif CCACGTCA. ELK4 belongs to the large E26 transformation-specific (ETS) family of TFs, which also include GABP, ETS, ETV, and ELF members, and binds to consensus sequence CCGGAAG. NFYB encodes one of the three subunits of nuclear transcription factor Y (NFY; the other being NFYA and NFYC) and binds with high affinity to CCAAT motif in the promoter region of target genes. While *Atf6a*, *Elk4*, and *Nfyb* are the most commonly found 12-h cycling TFs in mouse tissues, other family member/isoforms also often exhibit tissue-specific 12-h oscillations. For example, in mouse liver, bZIP TFs *Atf6a*, *Atf6b*, *Xbp1s*, *Creb3*, and *Creb3l2*, ETS TFs *Gabpa*, *Gabpb2*, *Elf1*, *Etv1*, *Etv5*, *Etv6*, *Elk4*, and *Ets2* all exhibit 12-h rhythms of expression. Thus, I hypothesize that ETS, bZIP, and NFY family of TFs form the core transcriptional architecture mediating the mammalian 12-h clock, even though the specific isoforms/members may vary in different tissues (Figure 3A). This tripartite transcriptional network is reminiscent of the E-box (BMAL1/CLOCK/PER/CRY), D-box (DBP/NFIL3), and RORE (ROR/REV-ERB)-binding TFs forming the core TTFL in mammalian circadian clock regulation (Takahashi, 2017).

As a first step toward testing this hypothesis, we studied the effects of hepatic XBP1s ablation on global 12-h transcriptome oscillation in mouse liver, and found prevalent impairment of the 12-h but not circadian mRNA oscillations in XBP1 liver-specific knockout mice (Pan et al., 2020). Specifically, XBP1s ablation abolished 54.5%, and dampened the amplitude of another 31.6% 12-h hepatic transcriptome, while having no effects on the core circadian clock in mouse liver (Pan et al., 2020). This observed independence of the majority of 12-h and circadian rhythms is consistent with previously uncovered largely intact 12-h rhythms of hepatic gene expression in *Bmal1* knockout and Clock^Δ19^ mutant mice (Zhu et al., 2017). Interestingly, among the direct XBP1s-targeted 12-h genes (which are defined as 12-h cycling genes with 12-h XBP1s chromatin recruitment to promoters and impaired 12-h oscillation in the absence of XBP1) are *Xbp1* itself, *Ire1α* (which alternatively spliced *Xbp1us*), *Atf6*, *Creb3*, *Creb3l2*, *Gabpa*, *Gabpb2*, *Elf1*, and *Nfyb*, which indicates the existence of an intricate network of transcriptional regulation within and among these three group of TFs (Figure 3A; Pan et al., 2020).

One remaining question is the identification of TFs mediating the negative feedback loop that is required for sustaining cell-autonomous oscillations of the 12-h clock. For the bZIP family of TFs, the top candidates are unspliced form of XBP1 (XBP1us) and ATF6β, both of which have been shown previously to antagonize XBP1s and ATF6α-mediated UPR (Yoshida et al., 2006; Thuerauf et al., 2007). For the ETS family, ETV6 is the most likely candidate as it has been found to strongly repress EBS-driven transcription (Mavrothalassitis and Ghysdael, 2000). Providing further support for XBP1us, ATF6β, and ETV6 participating in the negative feedback regulation of the 12-h clock is their near anti-phase 12-h mRNA oscillations with the transactivating counterparts in the same TF family (Figure 3B). As to the NFY family members, the situation could be more complicated. It has been previously shown that NFY can either act as an activator or a repressor (Zhu et al., 2012), an attribute likely arising from its unique ability to function as a pioneer factor to promote chromatin accessibility and affect subsequent TFs binding to adjacent sites (Oldfield et al., 2014). Therefore, there arises the tantalizing possibility that NFY can transcriptionally regulate the 12-h clock via facilitating the binding of bZIP and ETS family of TFs (both activator and repressors) via an assisted loading/facilitated repression mechanism, as previously described for BMAL1/ROR-mediated circadian clock control (Menet et al., 2014; Zhu et al., 2015). Future experiments are needed to test all the above hypotheses.

While all three families of TFs are expected to contribute to the transcriptional regulation of the mammalian 12-h clock collectively, with the roles of XBP1s and GABPA experimentally validated (Pan et al., 2020), could there be a hierarchy among them? Due to the lack of temporal transcriptome data from ETS/NFY knockout mice, we can only deduce the answer using ‘circumstantial evidence’. In mammalian circadian clock regulation, it is widely accepted that the E-box-binding TFs including BMAL1/CLOCK/PER/CRY form the ‘core’ TTFL. On the other hand, ROR/REV-ERB and DBP/NFIL3 are presumed to function as stabilizing or auxiliary transcriptional loops, and mainly regulate the amplitude of circadian gene oscillation (Takahashi, 2017). Coincidentally, *Bmal1* mRNA has the most restricted phase distribution in different tissues in baboon peaking from ZT12 to ZT16, among all key circadian clock TFs (Mure et al., 2018). If the relationship between hierarchy and phase distribution in the circadian clock control can be extrapolated to the 12-h clock regulation as well, then the ETS family of TFs are likely to be of the uttermost importance, as their phases are largely consistent in the four tissues with abundant 12-h transcriptome (liver, adrenal gland, MUS, and BAT) (Figure 3C). On the other hand, the phases of bZIP TFs *Atf6*, *Creb3*, and *Creb3l2*, as well as *Nfyb*, are disparate in the four tissues, with those in BAT and MUS anti-phase from those in liver and adrenal gland (Figure 3C). Intriguingly, the phases of these TFs are largely consistent with the phase distribution of the 12-h transcriptome in these four tissues (Figure 1C), suggesting that bZIP and NFY TFs may contribute more to the regulation of the 12-h clock output genes.

In sum, herein I propose that TFs belonging to ETS, bZIP, and NFY families are the core transcriptional mediators of the mammalian 12-h clock, with intricate positive feedforward and negative feedback loops connecting them (Figure 3A). In addition, it is worth noting that I am not ruling out the possibility that other TFs may also be implicated in the transcriptional regulation of the mammalian 12-h clock in a more tissue-specific manner (e.g. various KLF family members also exhibited DNA motif enrichment at 12-h cycling gene promoters and 12-h rhythms of gene expression in a limited number of tissues). As a last note, apparently, just like circadian rhythm, the mammalian 12-h clock is also subject to post-transcriptional and post-translational control (Zhu et al., 2017; Pan et al., 2020). Due to the page limit, I will not discuss these topics here.

## Conclusion

In this short perspective, I discussed our current knowledge of the prevalence, function, and regulation of the mammalian 12-h rhythms. Due to the very early stage of this field, many of the evidences presented in this article are still preliminary, and some of the conclusions are therefore somewhat speculative, and subject to modification with new experimental data. It is my sincere hope that this short article will attract more scientists into the nascent field of mammalian 12-h rhythms, as many more outstanding questions remain to be answered!

##  

*[This study was supported by the American Diabetes Association Junior Faculty Development Award 1-18-JDF-025 to B.Z.]*

**Edited by Jiarui Wu**
